# Detection of Waterborne and Airborne Formaldehyde: From Amperometric Chemosensing to a Visual Biosensor Based on Alcohol Oxidase

**DOI:** 10.3390/ma7021055

**Published:** 2014-02-11

**Authors:** Sasi Sigawi, Oleh Smutok, Olha Demkiv, Galina Gayda, Bohdan Vus, Yeshayahu Nitzan, Mykhailo Gonchar, Marina Nisnevitch

**Affiliations:** 1 Department of Chemical Engineering and Biotechnology, Ariel University, Ariel 40700, Israel; E-Mail: sasiyafit@walla.com; 2 The Mina and Everard Goodman Faculty of Life Sciences, Bar-Ilan University, Ramat-Gan 52900, Israel; E-Mail: nitzan.yeshayahu@biu.ac.il; 3 Department of Analytical Biotechnology, Institute of Cell Biology, Drahomanov Street 14/16, Lviv 79005, Ukraine; E-Mails: smutok@cellbiol.lviv.ua (O.S.); demkiv@yahoo.com (O.D.); galina_gayda@yahoo.com (G.G.); gonchar@cellbiol.lviv.ua (M.G.); 4 Lviv Polytechnic National University, S. Bandery Street 12, Lviv 79000, Ukraine; E-Mail: bogdan.vus@googlemail.com; 5 Institute of Applied Biotechnology and Basic Sciences, University of Rzeszow, Sokolowska Street 26, Kolbuszowa 36-100, Poland; E-Mail: mykhailo1952@gmail.com

**Keywords:** formaldehyde, microcomputer-based analyzer, chemosensing electrode, biosensor, alcohol oxidase, *Hansenula polymorpha*

## Abstract

A laboratory prototype of a microcomputer-based analyzer was developed for quantitative determination of formaldehyde in liquid samples, based on catalytic chemosensing elements. It was shown that selectivity for the target analyte could be increased by modulating the working electrode potential. Analytical parameters of three variants of the amperometric analyzer that differed in the chemical structure/configuration of the working electrode were studied. The constructed analyzer was tested on wastewater solutions that contained formaldehyde. A simple low-cost biosensor was developed for semi-quantitative detection of airborne formaldehyde in concentrations exceeding the threshold level. This biosensor is based on a change in the color of a solution that contains a mixture of alcohol oxidase from the yeast *Hansenula polymorpha*, horseradish peroxidase and a chromogen, following exposure to airborne formaldehyde. The solution is enclosed within a membrane device, which is permeable to formaldehyde vapors. The most efficient and sensitive biosensor for detecting formaldehyde was the one that contained alcohol oxidase with an activity of 1.2 U·mL^−1^. The biosensor requires no special instrumentation and enables rapid visual detection of airborne formaldehyde at concentrations, which are hazardous to human health.

## 1. Introduction

According to data of the International Agency for Research on Cancer (IARC) [1–5], formaldehyde (FA) is a toxic compound that can react with macromolecules in different biological systems and has mutagenic, immunogenic, allergenic and carcinogenic effects. FA has recently been described as one of the chemical mediators of apoptosis [6,7]. Interest in the possible role of FA in the pathogenesis of diabetic angiopathies, atherosclerosis and cardiovascular diseases has also increased in recent years. FA is currently considered to be the main cause for the sick building syndrome, which is defined as a set of symptoms associated with irritation of the upper air passages and eyes caused by harmful compounds (particularly FA) which are found in building materials, tobacco smoke, some medicinal preparations, *etc.* [8–10].

FA is a widespread chemical pollutant of water, air and soil. It can be found in food as well as in industrial and domestic sewage. FA was used in many industries and consumer products for decades as an antibacterial agent, antiseptic material, fumigant, bactericide, fungicide and insecticide. Use of FA in the food industry, where it serves as an antibacterial agent and as a preservative during food processing in order to prevent spoiling due to microbial contamination, is particularly important. Many industrial enterprises use it as a key chemical compound in organic synthesis and in combination with phenol for commercial production of composites, synthetic resins (urea-formaldehyde and phenol-formaldehyde, pentaerythritol and other compounds), adhesives, plastics, as a structural material in the automobile industry and for electrical engineering [11–16]. FA is also used in the textile industry. In particular, it is a component of a coupling agent that ensures the stability of fabric shapes. Wastewaters from many enterprises of different industries contain FA and phenols, which are hazardous to living organisms even at low concentrations. Fine cleaning and toxic substances control are therefore essential prior to the discharge of such wastewaters into the environment. FA is thus an important analyte in the control of many industrial goods, medicinal products and foodstuffs, as well as in environmental monitoring.

It is known that FA in indoor air causes several health problems. Exposure to airborne FA at 1–3 ppm leads to irritation of the eyes, nose and throat, and exposure to 10–20 ppm results in eye irritation and a burning sensation in the nose and throat as well as breathing difficulties that lead to coughing. However, many individuals cannot tolerate prolonged exposures to 4–5 ppm FA. Exposure to 50–100 ppm FA for a period of 5–10 min causes serious injury to the lower respiratory passages and chronic pulmonary obstruction [17].

The development of selective, highly sensitive, reliable and simple methods for fast and inexpensive FA detection is an urgent problem of analytical biotechnology and is necessary for environmental monitoring. Different approaches to analysis of this compound, including enzymatic and biosensor techniques [18–27], have been increasingly applied. A chemo- or biosensor (sensor analyzer) analyzer is an integral autonomic analytical device for quantitative or semi-quantitative analysis that uses a selective element, which is in direct contact with a physical transducer [23]. The selective element is usually placed/immobilized on the surface of the transducer. The important part of the biosensor is a microprocessor system (MS) that collects and processes analytical information.

The present work describes the development of: (1) A FA-selective chemosensing electrode that was integrated into an automatic amperometric analyzer for rapid quantitative determination of waterborne FA; (2) A simple low-cost biosensor for semi-quantitative detection of airborne FA in concentrations exceeding the threshold level. This biosensor is based on a change in the color of a solution containing a mixture of alcohol oxidase (AOX) from the yeast *Hansenula polymorpha*, horseradish peroxidase (HRP) and a chromogen, following exposure to airborne FA.

## 2. Results and Discussion

### 2.1. Amperometric Analyzer for Determination of Waterborne FA

#### 2.1.1. Development and Optimization of the Electronic Module of the Amperometric Chemosensor-Based Analyzer

**Figure 1.**
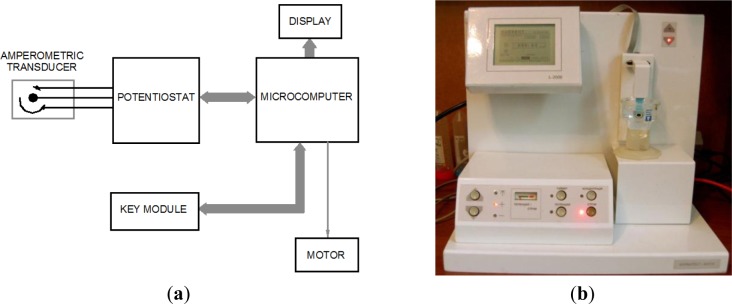
(**a**) Block diagram of the MC; and (**b**) Photograph of the chemosensor-based analyzer.

The developed analyzer (Figure 1) is composed of an amperometric transducer and a microcomputer (MC) for managing the process of analysis, processing and interpretation of information. The MC operates interactively, information is shown on a display, and a key module is used for control. Relocation of the amperometric transducer is performed by a stepper motor.

The following engineering problems were addressed in order to optimize the electronic circuits of the microcomputer-based system: matching the electric parameters of the transducer and the MC, definition of time parameters of the analysis stages, the potential, the amplification, the conditions of processing and the presentation of analytical results. Microcomputer-based software for interactive management and control of stages of experimental operations was developed and tuned. The following requirements were fulfilled: ensuring conditions for analysis and forming visual instructions in order to eliminate incorrect operations during analysis. It was established that a microprocessor system based on two microcomputers (for example, AT89C55WD microcontrollers) was reasonable.

#### 2.1.2. Characterization and Optimization of the Chemosensing Electrodes

A 4 mm diameter planar carbon electrode DRP-150, a 4 mm planar gold electrode DRP-C220AT (“DropSens”), and a 3.05 mm rod carbon electrode were used for construction of FA-selective chemosensors. All electrodes were modified with platinum black as a catalyst according to [28].

**Figure 2.**
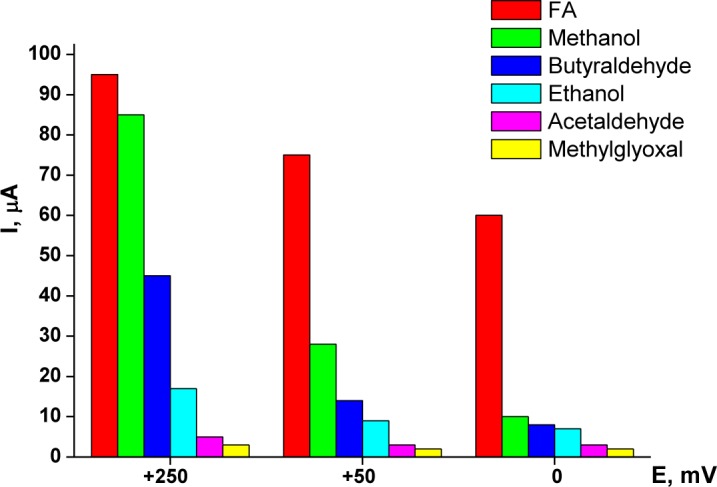
Amperometric response of the chemosensor based on a 3.05 mm rod platinized carbon electrode for various analytes at different working potentials: +250 mV, +50 mV, 0 mV *vs.* Ag/AgCl (20 mM analyte in 20 mM phosphate buffer (PB), pH 7.5).

Optimization of the working potential for chemoselective oxidation of FA also included a study of selectivity. During the preliminary stages of the research, it was found that the sensor exhibited a higher response (selectivity) for FA at a working potential of +250 mV *versus* Ag/AgCl, and a lower response for acetaldehyde, methylglyoxal, butyraldehyde, methanol and ethanol. A significant increase in the sensor’s selectivity was achieved at lower potentials of the working electrode (Figure 2).

As shown in Figure 2, decreasing the working potential leads to a decrease in the non-specific response to methanol, while retaining a high response to FA. Reduction of the sensor’s non-specific response to alcohols and some aldehydes at a low potential (0 mV *versus* Ag/AgCl) enables a rather selective determination of FA in real samples.

**Figure 3.**
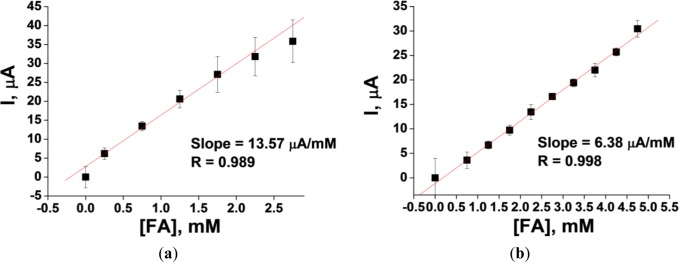
(**a**) Linearity test for the amperometric response of the 3.05 mm rod Pt-carbon electrode and 4 mm planar Pt-carbon electrode DRP-150 to FA; (**b**) Conditions: working potential 0 mV *vs.* Ag/AgCl, 20 mM PB, pH 7.5. Slopes of the lines (as sensitivity characteristics) and R (linear regression coefficients) are shown in the inserts.

The results of a linearity test for two platinized carbon electrodes—the 3.05 mm rod and the 4 mm planar electrode DRP-150*—*are presented in Figure 3. The linearity range for the two types of electrodes demonstrates significant differences. For example, the upper limit of linearity for the 4 mm planar Pt-carbon electrode DRP-150 (working surface area = 12.6 mm^2^) is 5 mM. This is considerably higher than the 2.5 mM limit of linearity for the 3.05 mm rod Pt-carbon electrode (working surface area = 7.3 mm^2^). However, the sensitivity of the former is three and half-fold lower than that of the latter (0.51 and 1.85 μA·mM^−1^·mm^2^, respectively).

The storage stability of the chemosensing electrodes was studied. The electrode’s catalytic surface did not lose its properties even after over one year of storage in a dry, dark place at room temperature.

#### 2.1.3. Determination of FA in Wastewater Samples

Further experiments showed that using a planar platinized gold electrode resulted in an essential improvement of the sensitivity to FA (3.76 μA·mM^−1^·mm^2^ *versus* 0.51 μA·mM^−1^·mm^2^ for the planar Pt-carbon electrode DRP-150). This electrode was therefore investigated further.

**Figure 4.**
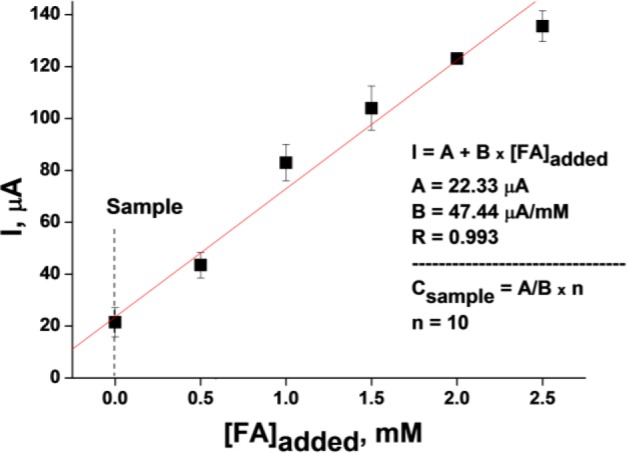
Amperometric determination of formaldehyde in a wastewater sample using a 4 mm planar Pt-gold electrode DRP-C220AT. Conditions: working potential 0 mV *vs.* Ag/AgCl, 20 mM PB, pH 7.5. Parameters of the line (“A” and “B”) are shown in the inserts: “A”—corresponds to the signal of the analyzed sample without addition of a FA standard, “B”—slope of the calibration curve, and “R”—linear regression coefficient, “*n*”—dilution of the tested sample. The calibration curve was obtained by adding aliquots of a FA standard solution to the analyzed sample.

Since FA is a chemically very active compound that can react with different components of the analyzed samples, analysis was performed in the multiple standard additions mode (Figure 4).

Analysis of a wastewater sample containing 4.90 ± 0.15 mM FA (estimated by a chemical method using chromotropic acid [29]) revealed a value of 4.71 ± 0.09 mM, which corresponds to a determination accuracy of 96%. Students’ *t*-test confirmed the non-significance of the difference between the results obtained by the two methods.

Although selectivity of the described chemosensor is lower compared to the formaldehyde dehydrogenase-based biosensors previously reported by us [25,27,30,31], it is more selective in comparison with an alcohol oxidase-based amperometric biosensor, which generates a positive output not only to formaldehyde, but an even higher signal to methanol and ethanol [32]. The potentiometric AOX-based biosensor [33] has a good selectivity to FA, but its sensitivity is lower than the currently described chemosensor. When compared to formaldehyde dehydrogenase-based sensors, the chemosensor is simpler in construction, because it does not require the enzyme and two co-enzymes (NAD+ and glutathione).

### 2.2. Semi-Quantitative Visual Biosensor for Airborne FA

A system that changes color upon contact with FA in a gaseous phase was constructed, in order to design a simple low-cost semi-quantitative biosensor for determination of airborne FA. The system was based on a membrane that separated the aqueous phase, which contains a chromogen and the enzymes AOX and HRP, and the FA-containing gaseous phase. FA diffused through the membrane to the aqueous phase and participated in the following chain process, which is catalyzed by the enzymes [34]:

$$H_{\text{2}}\mathrm{CO}+H_{\text{2}}O+O_{2}\overset{\mathrm{AOX}}{↔}\mathrm{HCOOH}+H_{\text{2}}O_{\text{2}}$$ (1)

$$2H-\mathrm{Chromogen}+H_{2}O_{2}\overset{\mathrm{HRP}}{↔}\mathrm{Red}\;\mathrm{Dye}+2H_{2}O$$ (2)

**Figure 5.**
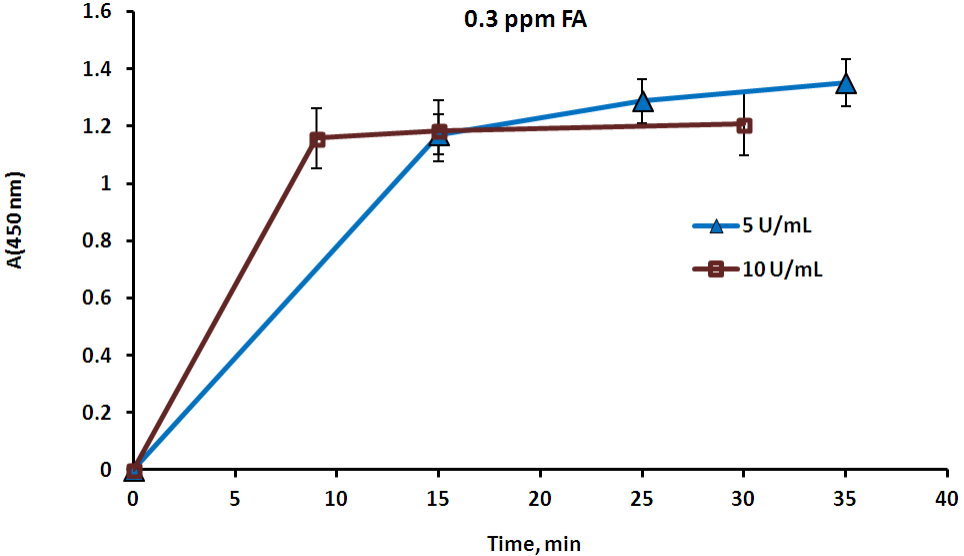
Color development in the membrane devices containing 3.5 mg·mL^−1^ of HRP, 2 mg·mL^−1^ of chromogen and 5 or 10 U·mL^−1^ of AOX. The devices were exposed to airborne FA at a concentration of 0.3 ppm, and the color intensity was measured at various time periods starting from the color development point.

The rate of color appearance in the second reaction actually depended on the FA concentration in the aqueous phase, which in turn depended on the FA concentration in the gaseous phase. Several experiments were carried out using a closed vessel that contained a layer of the FA aqueous solution at a known concentration, in order to study this effect. Henry’s law enables the calculation of the airborne FA concentration [35], which was also proven by measurement with the FA gas detector. Membrane devices containing various concentrations of AOX, HRP and a chromogen were placed above the aqueous layer, in contact solely with the gaseous phase. Reactions (1) and (2) took place upon diffusion of FA through the membrane into the aqueous phase, and the time of the clearly visually detectable appearance of color was registered. The membrane devices were then removed from the vessel, opened, and the color intensity was measured spectrophotometrically at the appropriate wavelength [36]. The color intensity was found to correspond to an absorbance A (450 nm) range from 1 to 1.2 in all cases. The clearest results were obtained when HRP was used at a concentration of 3.5 mg·mL^−1^ (1000 U·mL^−1^) and the chromogen—at a concentration of 2 mg·mL^−1^. According to the Material Safety Data Sheet (MSDS), the threshold level value (TLV) of airborne FA is 0.3 ppm [37]. Three FA rates, 0.1, 0.3 and 18.5 ppm, were therefore chosen as fixed points for evaluating the reaction time. The first point corresponds to the safe FA level, the second to the maximum permissible level and the third is considered to be a level dangerous for humans. An optimal biosensor would react immediately to the dangerous FA level, would react moderately to the permissible level and would not react at all to the safe level. The effect of AOX activity on the time of color development was studied in order to achieve this aim (Figure 5). The reaction time was found to be inversely dependent on the AOX concentration, and once the color developed it remained stable for at least half an hour (Figure 5).

**Figure 6.**
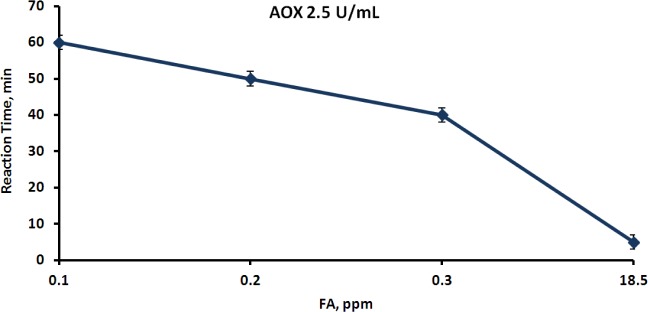
Time of color development in the membrane devices containing HRP—3.5 mg·mL^−1^, chromogen—2 mg·mL^−1^ and AOX—2.5 U·mL^−1^. The devices were exposed to airborne FA at various concentrations and the reaction time was registered.

In the next stage, the effect of the airborne FA concentration on the time of color development was studied. For this aim, the AOX concentration in the membrane devices was kept constant, and the devices were exposed to various concentrations of airborne FA (Figure 6).

As shown in Figure 6, the time of color development depended on the FA concentration in the gaseous phase, and the higher the FA concentration, the shorter was the reaction time. This fact can easily be explained by the dependence of the FA diffusion rate through the membrane from the gaseous into the aqueous phase on the airborne FA concentration.

**Figure 7.**
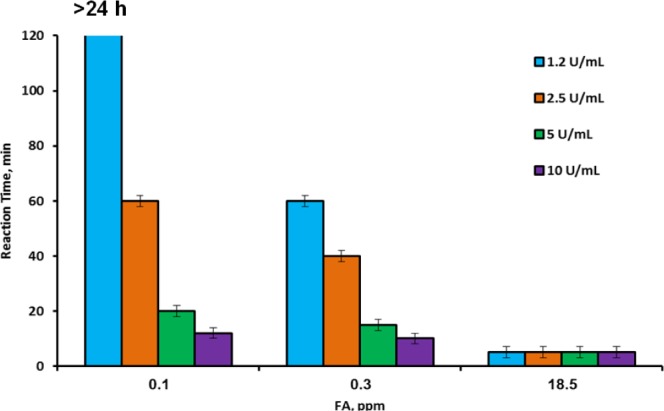
Time of color development in the membrane devices containing 3.5 mg·mL^−1^ of HRP, 2 mg·mL^−1^ of chromogen and various concentrations of AOX. The devices were exposed to airborne FA at various concentrations, and the reaction time was registered.

Experiments with varying FA and AOX concentrations were performed in order to determine and choose conditions for a selective reaction of the membrane device to safe and dangerous FA concentrations in the gaseous phase. The results of this series are presented in Figure 7. As shown in Figure 7, there was almost no difference between the color development time under exposure to 0.1 and 18.5 ppm at high AOX concentrations (5 to 10 U·mL^−1^)—the color appeared already after 12–20 min in the first case and after several minutes in the second. With the medium AOX concentration (2.5 U·mL^−1^), the reaction times to high and low FA levels were clearly distinguished—the device changed color within 3–5 min when exposed to 18.5 ppm and within 40 min when exposed to 0.3 ppm. However, the reaction to 0.1 ppm took place after 60 min so that the difference between the safe and the threshold levels was not sufficiently clear. The results for the lowest applied concentration of AOX (1.25 U·mL^−1^) were very satisfactory. The device reacted very quickly to the dangerous 18.5 ppm concentration of FA, and very slowly (1 h) to the TLV concentration, but did not react at all to the safe 0.1 ppm concentration (the color did not develop during 24 h of exposure). This result enabled unambiguous differentiation between safe, threshold and dangerous concentrations of airborne FA. It also enabled reaching the conclusion that the proposed membrane device could be applied as a semi-quantitative biosensor for detection of FA in indoor air. The simplicity of the device opens prospects for its wide application in workstations related to the use of FA, from workshops to laboratories. The device showed good reproducibility and reliability of the results. In the experiments in which light absorption was measured at the chosen time periods, the relative standard errors (δ) did not exceed 3%. In the experiments in which the time of visual color development was measured, the relative standard errors varied from 20% for short periods (several minutes) to 2% for long periods (more than an hour).

The proposed biosensor for semi-quantitative determination of airborne FA has several advantages over existing instrumental methods of detection, such as chromatography or selective gas analyzers [37]. The biosensor does not require any instrumental support, but at the same time supplies an almost immediate reaction to hazardous levels of FA, when the developed visual signal can be clearly observed. The only disadvantage of this biosensor is its irreversibility, because once developed, the color does not disappear. However, this issue is compensated by the low cost and simplicity of the device, which can be produced wholesale and can be stored for a long time, since AOX and HRP are known for their high stability [38,39].

## 3. Experimental Section

### 3.1. Materials

DEAE-Toyopearl 650M was purchased from Toyo Soda (Tokyo, Japan); phenylmethylsulfonyl fluoride (PMSF), EDTA and Triton X-100 were obtained from Sigma (Deisenhofen, Germany). Ethanol absolute was purchased from Riedel-de Haën (Seelze, Germany); hexachloroplatinum(IV)-acid-hexahydrate and methanol were obtained from Merck-Schuchardt (Hohenbrunn, Germany); HRP with a specific activity of 250–330 units per 1 mg solid (RZ 3.0) and *o*-dianisidine were purchased from Sigma Aldrich Chemie (Steinheim, Germany).

All chemicals were of analytical reagent grade and all solutions were prepared using high performance liquid chromatography (HPLC)-grade water. The FA solution (1 M) was prepared by hydrolysis of the corresponding amount of para-formaldehyde in water (300 mg; 10 mL water) under heating the suspension in a sealed ampoule at 105°C for 6 h.

### 3.2. Isolation of AOX from Hansenula polymorpha

A mutant strain of the thermotolerant methylotrophic yeast *H. polymorpha* C-105 (*gcr1 catX*) selected in the Institute of Cell Biology (Lviv, Ukraine) [40] was used as a producer of AOX. This strain has impairment in glucose catabolite repression of AOX synthesis, is catalase-defective, and has the ability to over-produce AOX in glucose medium. Mutant *H. polymorpha* C-105 cells were cultivated in flasks on a shaker (200 rpm) at 30°C up to the middle of the exponential growth phase (~24 h) in medium containing (g/L) the following: glucose, 10; (NH_4_)_2_SO_4_, 3.5; KH_2_PO_4_, 1.0; MgSO_4_·7H_2_O, 0.5; CaCl_2_, 0.1; yeast extract, 3.0. The pH of the medium was adjusted to 5.5 with KOH. After washing, freshly grown cells (about 15 g wet weight) were re-suspended in two volumes of 0.05 M phosphate buffer, pH 7.5. The cells were disrupted in the presence of glass beads (*d* = 0.45–0.50 mm) in a planetary disintegrator at 1000 rpm (*r*_av_ 10 cm) and 4°C for 6 min. Whole cells and cell debris were removed by centrifugation at 15,000 *g* (*r*_av_ 8 cm) for 40 min. The supernatant cell-free extract was used for isolation of AOX.

Highly purified AOX was isolated by means of a two-step ammonium sulfate fractionation (at 40% and 60% of saturation), followed by ion exchange chromatography on DEAE-Toyopearl 650M [41]. The first stages of the purification process were carried out in the presence of protease inhibitors (1 mM EDTA and 0.4 mM phenylmethylsulfonyl fluoride, PMSF). The final preparation of the enzyme had a specific activity of 30 U per mg protein and was homogeneous during electrophoresis in a polyacrylamide gel in the presence of SDS. AOX preparations were stored as a suspension in 70% saturated ammonium sulfate (with an activity 200 U·mL^−1^ of suspension) at −10°C without any remarkable decrease in activity over a period of several months. Before use, the enzyme suspension was centrifuged and the enzyme precipitate was dissolved in 0.1 M phosphate buffer, pH 7.6.

### 3.3. Analysis of AOX Activity

AOX activity was determined by the rate of hydrogen peroxide formation in reaction with methanol as monitored by the peroxidative oxidation of *o*-dianisidine in the presence of HRP [42]. The millimolar extinction coefficient of the colored product in an acidic solution (2.5 M HCl) at 525 nm was 13.38 mM^–1^·cm^–1^. Protein concentration was determined by the Lowry method.

### 3.4. Preparation of an Amperometric Chemosensor for Waterborne FA Determination

The chemosensors were evaluated by constant-potential amperometry in a three-electrode configuration with an Ag/AgCl reference electrode, a Pt-wire counter electrode, and working electrodes—the 3.05 mm diameter carbon rod (type RW001, Ringsdorff Werke, Bonn, Germany) sealed in a glass tube using epoxy glue, the 4 mm planar carbon electrode DRP-150, and the planar gold electrode DRP-C220AT from DropSens [Llanera (Asturias), Spain].

Platinized electrodes were prepared by electrochemical Pt deposition from a 2% solution of H_2_PtCl_6_ according to [28]. Calibration was performed by stepwise addition of a standard FA solution.

### 3.5. Design of a Semi-Quantitative Visual Membrane Biosensor Device for Detection of Airborne FA

The membrane biosensor device for detection of airborne FA was prepared based on dialysis tubes (Sigma-Aldrich) containing 1 mL of a solution containing 3.5 mg·mL^−1^ of HRP with a specific activity of 250–330 units (*RZ 3.0*) per 1 mg solid, 2 mg·mL^−1^ *o*-dianisidine as a chromogen (both purchased from Sigma Aldrich Chemie (Steinheim, Germany)), and 1.2–10 U·mL^−1^ AOX in 0.05 M sodium phosphate buffer, pH 7.5. The device was exposed to various concentrations of airborne FA—from 0.1 to 18.5 ppm, obtained by saturating the gaseous phase with FA vapors which were in equilibrium with aqueous FA solutions of various concentrations (0.54–97.5 mM). The airborne FA concentration was calculated based on Henry’s law, where the Henry constants were calculated by the following equation [35]:

$$\ln H_{A}(\mathrm{atm}\cdot L\cdot {\mathrm{mol}}^{-1})=-1641.3/T-3.089$$ (3)

and also tested in the range of 0.1–0.6 ppm by the FA Gas Detector (Model FP-40 Riken Keiki, Japan). The FA solutions were placed in closed prism-form glass vessels supplied with stoppered flat covers, which enabled quick opening and closing of the vessel, in order to achieve FA equilibrium between the gaseous and aqueous phases. The above-described membrane devices were placed in the vessels on racks, so that the device was in contact solely with the gaseous phase. The time of the color development was registered, and immediately thereafter the devices were opened and the color intensity was measured using the UV-Vis Cary-50 (Varian, Australia) spectrophotometer.

### 3.6. Statistics

The results obtained from at least three independent experiments fulfilled with duplicates were statistically analyzed by Anova single factor analyses or by Students’ *t*-test. The difference between the results was considered significant if the *p*-value was less than 0.05. Relative standard errors were calculated on the basis of the Descriptive Statistics data.

## 4. Conclusions

An amperometric analyzer with a chemosensing catalytic element was developed for the assay of waterborne and airborne formaldehyde. The chemosensing recognition element is based on platinum-dependent oxidation of FA. The sensing electrode is very stable (no inactivation after over one year of use) and is satisfactorily selective (due to using a low working potential). Use of commercial screen-printed electrodes enables simplifying the preparation procedure of the chemosensing element and results in a high reproducibility of the FA analysis. The proposed universal optimized electronic module of the amperometric analyzer can be adapted to the analysis of other analytes by integrating the corresponding sensing element into the biosensitive layer. The developed amperometric analyzer with a chemosensing catalytic element for quantitative determination of FA was tested on a sample of wastewater. A high recovery of the target analyte (96%) demonstrates the possibility of using the constructed analyzer for determination of FA in real samples.

A semi-quantitative visual membrane biosensor device was proposed for detection of airborne FA. The device is based on a change in the color of a chromogen in the presence of AOX and HRP, triggered by FA, where the time of color development depends on the FA concentration in the gaseous phase. The developed biosensor demonstrates high selectivity towards safe and dangerous concentrations of airborne FA and can serve as an effective and rapidly reactive indicator of high airborne FA concentrations.
